# Toric Versus non-toric intraocular lenses for pre-existing corneal astigmatism: a systematic review and meta-analysis

**DOI:** 10.3389/fmed.2026.1777267

**Published:** 2026-03-23

**Authors:** Le-Han Hao, Cheng Pei

**Affiliations:** Department of Ophthalmology, The First Affiliated Hospital of Xi'an Jiaotong University, Xi'an, Shaanxi, China

**Keywords:** cataract surgery, corneal astigmatism, non-toricintraocular lenses, systematic review, toric intraocular lens

## Abstract

**Background:**

Preoperative corneal astigmatism is prevalent among cataract patients and has a negative effect on uncorrected distance visual acuity. Toric IOLs are commonly used to correct astigmatism and clinical trials and expert consensus have reported advantages as compared to non-toric IOLs, but the degree and the consistency of these improvements remain to be more clearly defined.

**Methods:**

Clinical studies comparing toric and non-toric IOL implantation in cataract patients with corneal astigmatism were identified in PubMed and Web of Science. The primary outcome was postoperative residual refractive astigmatism; secondary outcomes were UDVA, corrected distance visual acuity, the proportion of eyes with residual refractive cylinder ≤ 0.5 D, and postoperative spherical equivalent. Effect estimates were pooled as standardized mean differences, mean differences or odds ratios along with 95% confidence intervals.

**Results:**

A total of twelve studies qualified for inclusion. Toric IOLs yielded lower residual refractive astigmatism (SMD = −1.03, 95% CI −1.16 to −0.90) and better UDVA (SMD = −0.91, 95% CI −1.07 to −0.76) than non-toric IOLs. More eyes with toric IOLs achieved residual cylinder ≤ 0.5 D (OR = 3.31, 95% CI 2.44 to 4.48). The differences in spherical equivalent were small (MD = 0.07 D, 95% CI 0.02 to 0.13) and are likely to be of limited clinical relevance.

**Conclusions:**

In patients with corneal astigmatism, toric IOLs provide more precise astigmatic correction and better uncorrected distance vision. These pooled effect sizes refine the current consensus and may assist refractive planning in cataract surgery.

## 1 Introduction

Cataract surgery is increasingly regarded as a refractive procedure, with patients expecting not only restoration of corrected distance visual acuity but also good uncorrected distance visual acuity (UDVA) and reduced dependence on spectacles (1). Large preoperative series have shown that a substantial proportion of eyes scheduled for cataract surgery have clinically significant corneal astigmatism (1–4), which can compromise uncorrected distance visual acuity if left uncorrected. Residual refractive astigmatism after surgery may therefore lead to suboptimal visual performance and postoperative dissatisfaction and has become an important target for systematic management in contemporary cataract practice.

Several intraoperative strategies are available to address corneal astigmatism at the time of cataract surgery, including incision placement on the steep meridian, corneal relaxing or arcuate keratotomy and implantation of toric intraocular lenses (IOLs) (5). Toric lenses offer a lens based solution that can provide a relatively stable and predictable reduction in postoperative refractive cylinder and are less influenced by corneal wound healing than corneal incision based approaches (6). Clinical success, however, depends on accurate identification of the astigmatic axis, precise intraoperative alignment and adequate postoperative rotational stability, because even small misalignments can substantially reduce the effective astigmatic correction (7). Recent clinical and experimental work has therefore focused on rotational behavior, alignment methods and biometric factors that may affect refractive accuracy (8–10).

In recent years, randomized trials and observational cohort studies have compared toric and non-toric lenses in cataract patients with pre-existing corneal astigmatism. In general, these studies have shown that toric lenses reduce residual refractive cylinder and improve uncorrected distance visual acuity in appropriately selected eyes, although the magnitude of benefit and the impact on other outcomes vary between reports (11, 12). Previous synthesis level evidence has also demonstrated advantages of toric lenses in terms of uncorrected distance visual acuity, lower residual refractive astigmatism and higher spectacle independence, while highlighting notable diversity across studies that may be related to differences in baseline astigmatism magnitude, lens models, surgical techniques and outcome assessment (13, 14).

Several clinically relevant questions remain. The range of low corneal astigmatism in which toric lenses provide a meaningful advantage over non-toric options is still debated, particularly in light of concepts such as total corneal astigmatism and newer measurement and calculation techniques (15, 16). In addition, developments in image guided alignment, biometry and toric calculators may have altered expected outcomes compared with older reports, making it important to update and refine the available evidence with a focus on refractive cataract surgery endpoints such as postoperative residual refractive astigmatism, uncorrected and corrected distance visual acuity and refractive predictability (17).

Against this background, we performed a systematic review and meta-analysis to compare toric and non-toric IOL implantation in cataract patients with pre-existing corneal astigmatism. Rather than re-examining whether toric lenses are superior, the primary objective was to quantify the magnitude of their refractive and visual benefits and to summarize key outcomes across contemporary clinical studies, in order to refine existing consensus and provide updated quantitative evidence to support lens selection and refractive planning in routine practice.

## 2 Materials and methods

### 2.1 Literature search strategy

A systematic literature search in the PubMed and Web of Science databases was conducted to find eligible studies evaluating toric and non-toric IOLs in cataract patients with corneal astigmatism. The search included all records available until the date of the final search.

The following keywords with Boolean operators (“AND”, “OR”) were adopted for the literature search in PubMed and Web of Science: toric IOLs, non-toric IOLs, cataract surgery and astigmatism. Then the reference lists of the related studies in English were manually screened for a further identification.

### 2.2 Eligibility criteria

The following criteria were applied to consider studies for inclusion. The study involved adult patients with corneal astigmatism who were scheduled for cataract surgery. Eligible studies were those comparing treatment with toric IOLs vs. non-toric IOLs. The following postoperative outcomes should be reported from the included studies: residual postoperative refractive astigmatism, UDVA, CDVA, cases of eyes with residual refractive cylinder ≤ 0.5 D, and postoperative spherical equivalent. Both RCTs and observational cohort studies were eligible for inclusion.

Studies were excluded if any of them did not make comparisons between toric and non-toric IOLs, or if no quantitative outcome data were available for extraction for meta-analysis. Case reports, review articles, conference abstracts, editorials and letters were excluded as well. In addition, duplicate publications and studies with overlapping patient populations were excluded to avoid data redundancy.

### 2.3 Study selection process

**Figure 1:**
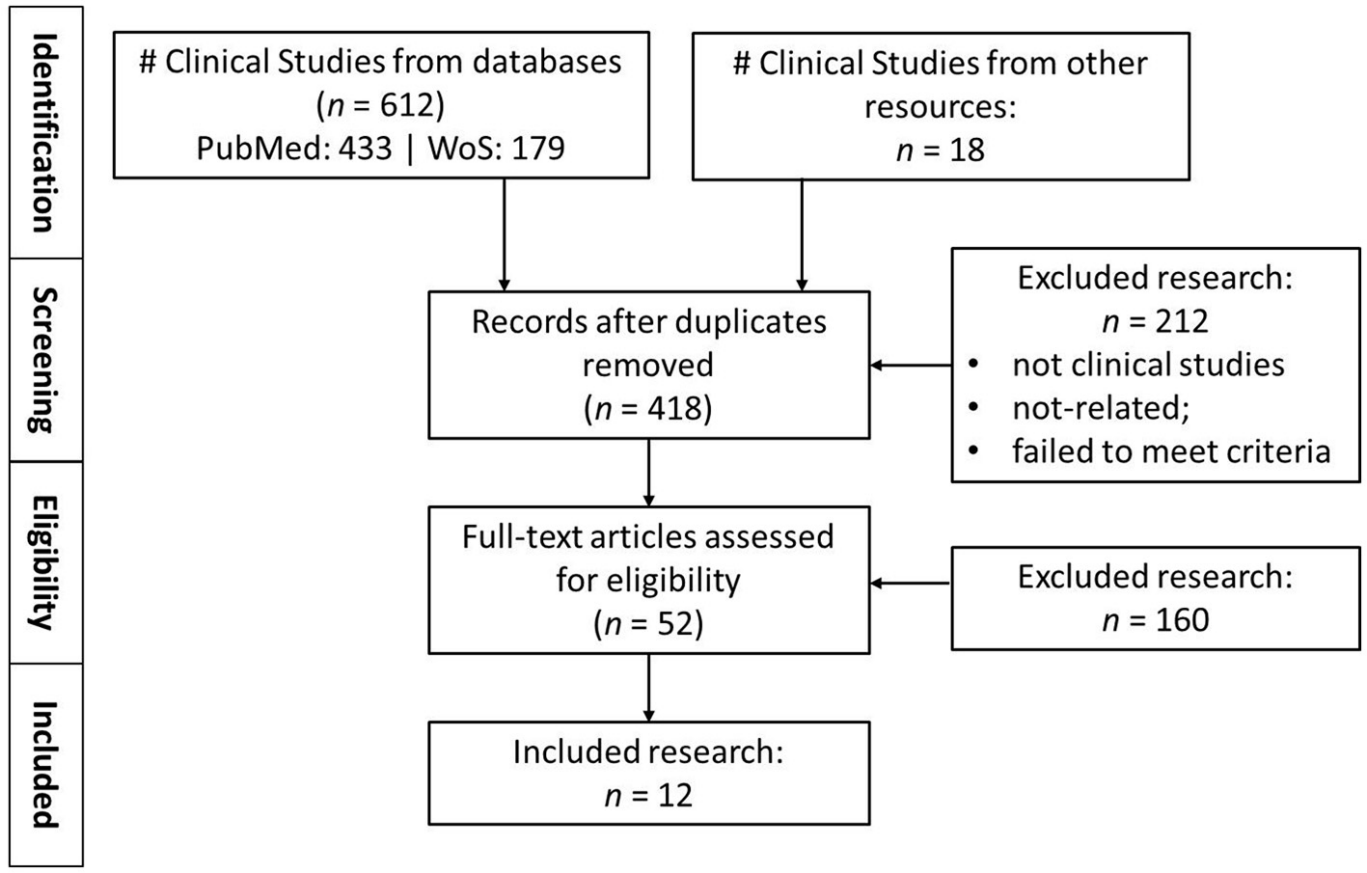
Flowchart of the search process under the PRISMA guidelines.

All the retrieved records were independently screened by two reviewers according to titles and abstracts. Full text articles were then evaluated when inclusion or exclusion could not be determined from the abstract alone. Any disagreements were resolved through discussion and consensus. The process of study selection is depicted in a PRISMA (Preferred Reporting Items for Systematic Reviews and Meta-Analyses) flow diagram (Figure 1). The characteristics of the included studies are summarized in Table 1.

**Table 1:** Demographic features of the population in the included studies.

**Study ID**	**First author**	**Publication year**	**Country**	**Study design**	**Follow-up duration**
1	Anand et al. (1)	2025	India	Cohort study	2 years
2	Bissen-Miyajima et al. (2)	2015	Japan	Cohort study	1 year
3	Ding et al. (3)	2022	China	Cohort study	3 months
4	Gundersen et al. (4)	2020	USA	Cohort study	30 months
5	Holland et al. (5)	2010	USA	Cohort study	1 year
6	Liu et al. (6)	2021	China	Cohort study	3 months
7	Nagpal et al. (7)	2015	India	RCT	6 months
8	Park et al. (8)	2011	Korea	Cohort study	6 months
9	Shin et al. (9)	2021	Korea	Cohort study	2 months
10	Visser et al. (10)	2014	The Netherlands	RCT	6 months
11	Wang et al. (11)	2023	China	Cohort study	3 months
12	Yamauchi et al. (12)	2018	Japan	Cohort study	2 months

### 2.4 Data extraction

Two reviewers extracted data in duplicate using a standardized data extraction form. Study characteristics, sample size as number of eyes analyzed, patient demographics, type of IOLs implanted, and follow-up period were recorded. Postoperative outcome measures were extracted as reported by the original studies.

Results were not reported consistently within or across studies. When outcomes were presented in multiple formats, the data were converted to comparable metrics to facilitate quantitative synthesis. If outcome data could not be reliably extracted, they were excluded from the analysis.

### 2.5 Outcome measures

The main outcome of interest was postoperative residual refractive astigmatism as a continuous variable. Secondary outcomes were UDVA (in logMAR) and CDVA (in logMAR), the postoperative spherical equivalent in diopters, and the proportion of eyes with a postoperative residual refractive cylinder ≤ 0.5 D as a dichotomous variable. In this meta-analysis, we focused on the magnitude and consistency of the differences between toric and non-toric intraocular lenses across these outcomes, in order to better characterize the clinical relevance of toric IOL implantation in contemporary cataract practice.

### 2.6 Statistical analysis

The statistical analysis was conducted with RevMan software (version 5.4, Cochrane Collaboration, Copenhagen, Denmark). For continuous outcomes reported using different scales in different studies, SMDs with 95% CIs were calculated. Standardized mean differences were chosen for visual acuity outcomes because different studies reported these measures using non-identical scales, and this approach allows results to be combined when the same construct is measured using different units. For the analysis of the proportion of eyes with a postoperative residual refractive cylinder ≤ 0.5 D as a dichotomous variable, OR with 95% CI was adopted. The heterogeneity was evaluated using the χ^2^ test and quantified with the *I*^2^ statistic, and the statistical significance was determined as a two-sided *p* value < 0.05. A random effects model with inverse variance weighting was applied for all meta-analyses, because some clinical and methodological heterogeneity between studies was expected. When appropriate, subgroup or sensitivity analyses were considered to explore sources of heterogeneity and to assess the robustness of pooled estimates.

## 3 Results

### 3.1 Study selection

After removal of duplicates, 418 records were screened according to titles and abstracts. Of those, 366 were excluded due to irrelevance or not meeting inclusion criteria. Fifty-two full text articles were evaluated for potential inclusion, 40 were excluded for various reasons including no direct comparison between toric and non-toric IOL, too few or not relevant outcome data, or overlapping study populations. In the end, 12 studies were eligible for inclusion in the meta-analysis. The selection of studies is shown in the PRISMA flow chart (Figure 1) (12, 18–28).

### 3.2 Study characteristics and risk of bias assessment

**Figure 2:**
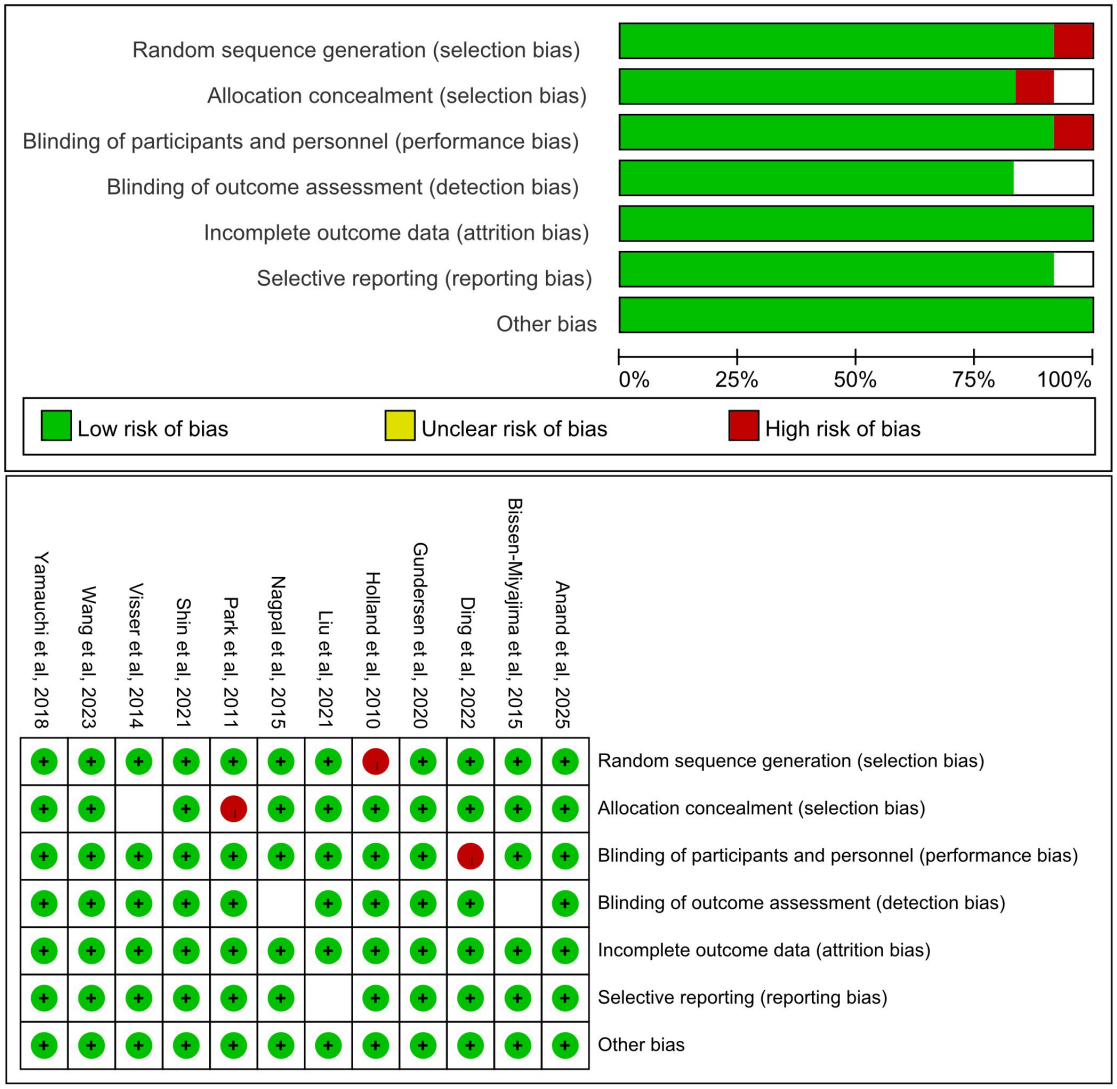
Risk of bias assessment of the included randomized controlled trials using the Cochrane Risk of Bias tool.

Included were both RCTs and cohort studies that compared toric and non-toric IOLs in cataract surgery patients with pre-existing corneal astigmatism. The follow-up periods ranged from the early postoperative term to mid-term. Quality assessment risk of bias was assessed using the Cochrane Risk of Bias tool among the clinical studies, as illustrated in Figure 2. Across the included trials and cohorts, eyes were required to have at least mild preoperative corneal astigmatism. Where the threshold was reported, the lower limit for keratometric cylinder ranged between about 0.75 D and 1.00 D. Most studies enrolled eyes with baseline corneal astigmatism up to approximately 2.50 D to 3.00 D. Some reports focused on eyes with relatively low astigmatism, usually defined as 1.50 D or less, whereas others covered a broader astigmatic spectrum as defined by the original authors. This variation in baseline astigmatism levels is considered a relevant source of clinical heterogeneity in the present analysis.

### 3.3 Postoperative residual refractive astigmatism

**Figure 3:**
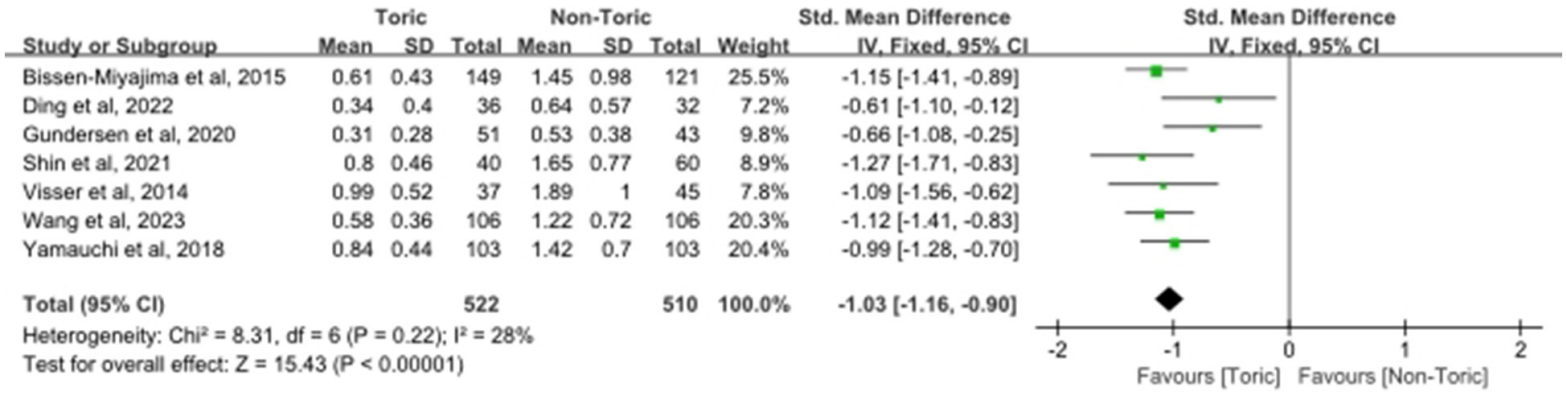
Forest plot of postoperative residual refractive astigmatism.

Postoperative manifest refractive cylinder was used to define residual refractive astigmatism. Seven articles including 510 eyes contributed data to this analysis. Toric IOL implantation was associated with significantly lower postoperative residual refractive astigmatism compared with non-toric IOLs (SMD = −1.03, 95% CI −1.16 to −0.90; Z = 15.43, *P* < 0.00001; Figure 3). Although heterogeneity was only moderate (χ^2^ = 8.31, df = 6, *P* = 0.22; *I*^2^ = 28%), the direction of effect consistently favored toric IOLs across individual studies, indicating a clear and consistent reduction in residual refractive cylinder.

### 3.4 Postoperative UDVA

**Figure 4:**
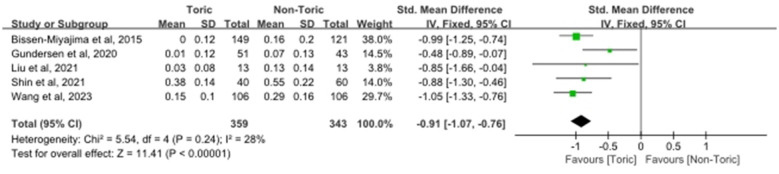
Forest plot of UDVA.

Five studies involving a total of 343 eyes were included for the analysis of postoperative UDVA. The pooled estimate showed lower postoperative UDVA (logMAR) in the toric IOL group compared with the non-toric IOL group (SMD = −0.91, 95% CI −1.07 to −0.76; *Z* = 11.41, *P* < 0.00001; Figure 4). Heterogeneity was low to moderate (χ^2^ = 5.54, df = 4, *P* = 0.24; *I*^2^ = 28%). Overall, these findings suggest a clinically relevant improvement in unaided distance visual performance with toric IOL implantation in this patient population.

### 3.5 Postoperative CDVA

**Figure 5:**
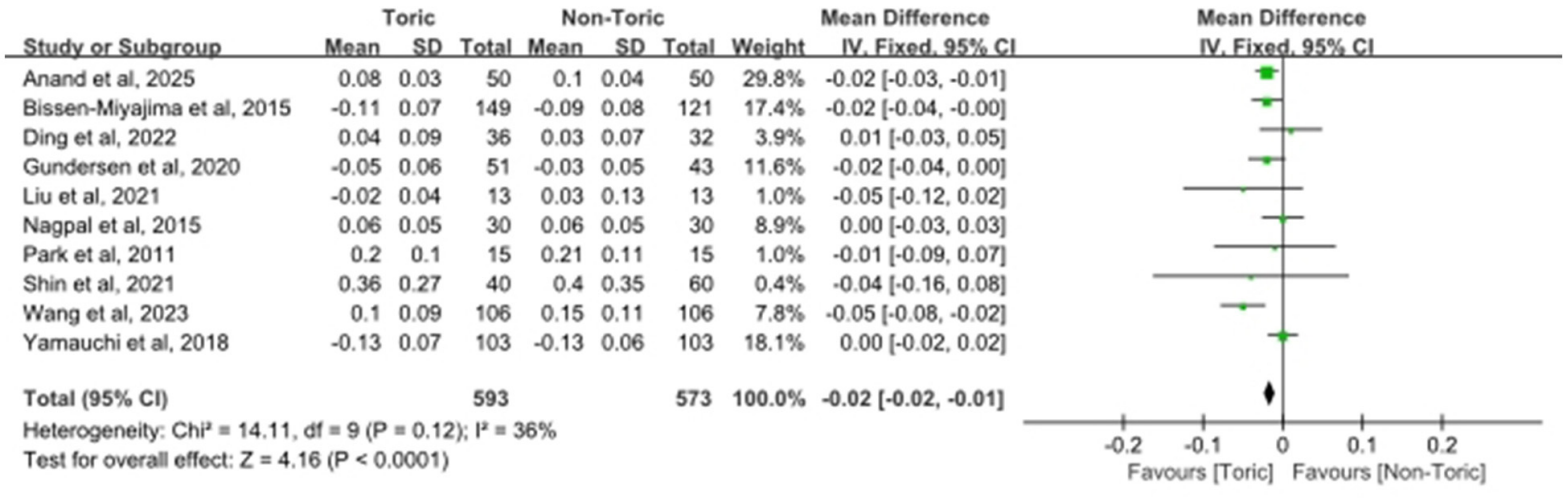
Forest plot of CDVA.

Eight studies involving 573 eyes in total were included for the analysis of postoperative CDVA (in logMAR). As presented in Figure 5, there was a statistically significant difference in the post-operative CDVA between the toric IOLs and the non-toric IOLs, favoring the toric IOLs (MD = −0.02 logMAR, 95% CI −0.02 to −0.01, *Z* = 4.16, *P* < 0.0001). Moderate heterogeneity was found among the included studies (χ^2^ = 14.11, df = 9, *P* = 0.12; *I*^2^ = 36%). The estimates of individual studies were scattered on both sides of the line of no effect, and the CIs overlapped.

### 3.6 Proportion of eyes with residual refractive cylinder ≤ 0.5 D

**Figure 6:**
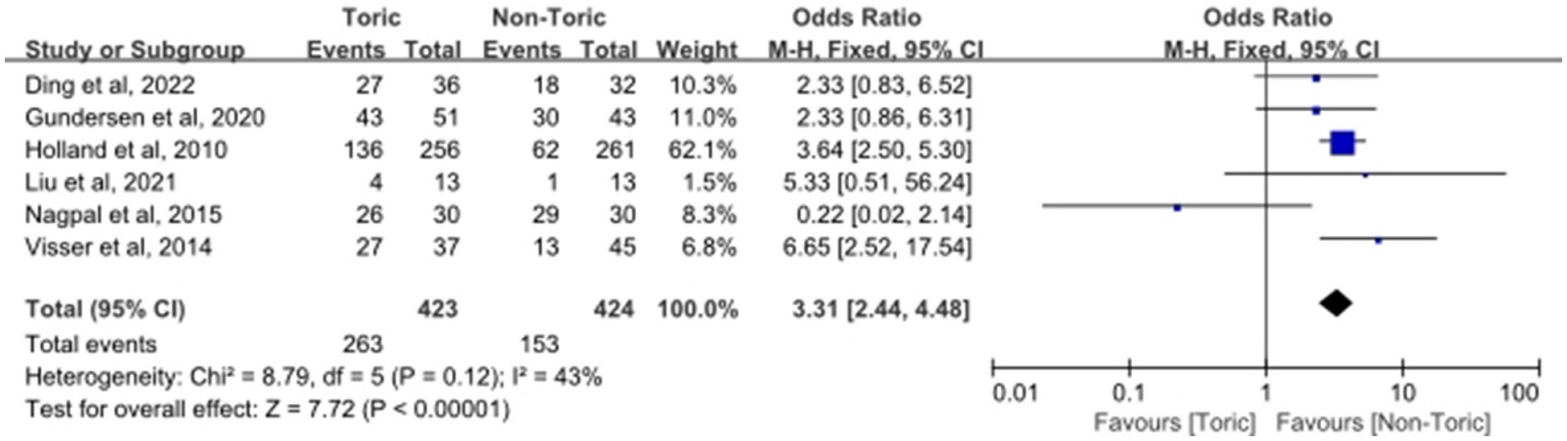
Forest plot of the proportion of eyes with residual refractive cylinder ≤ 0.5 D.

The proportion of eyes attaining a postoperative residual refractive cylinder ≤ 0.5 D was investigated as a categorical result. Five reports on 687 eyes were considered. As shown in Figure 6, a significantly higher percentage of eyes achieved this end point in the toric IOLs group compared with the non-toric group (OR = 3.31, 95% CI 2.44 to 4.48; *Z* = 7.72, *P* < 0.00001). While moderate heterogeneity was detected among the studies (χ^2^ = 8.79, df = 5, P = 0.12; *I*^2^ = 43%), the OR from multiple studies also favored the toric IOL group.

### 3.7 Postoperative spherical equivalent

**Figure 7:**
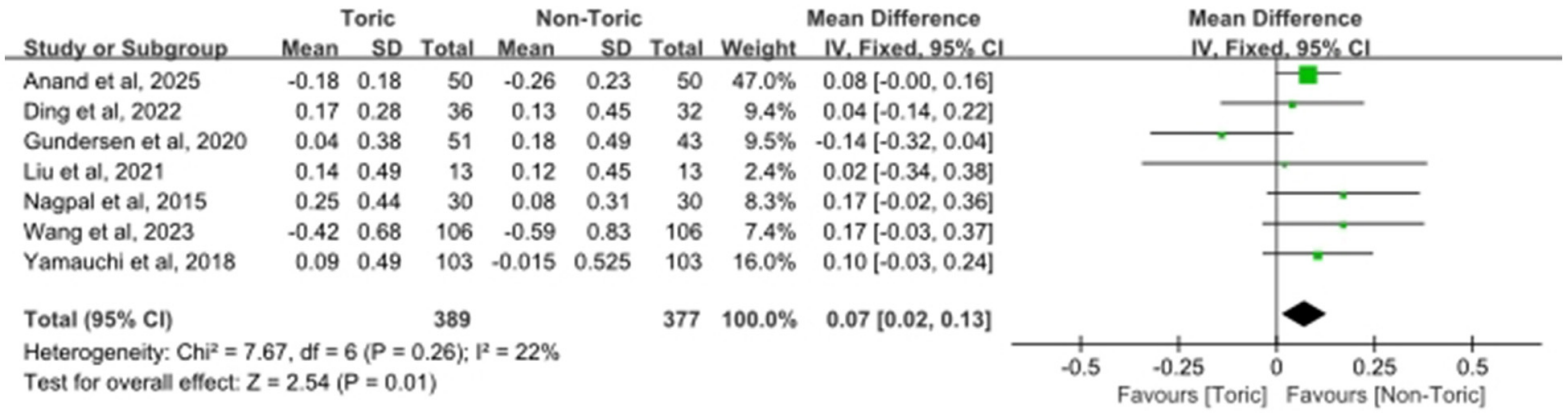
Forest plot of postoperative spherical equivalent.

Out of all the studies, seven reports which included 377 eyes were analyzed for the outcome postoperative spherical equivalent. Meta-analysis showed a minor but statistically significant difference in postoperative spherical equivalent between toric and non-toric IOLs (MD = 0.07 D, 95% CI 0.02 to 0.13; *Z* = 2.54, *P* = 0.01) as shown in Figure 7. Moderate heterogeneity was found among these 7 studies (χ^2^ = 7.67, df = 6, *P* = 0.26; *I*^2^ = 22%). Individual study estimates were scattered on both sides of the line of no effect, with overlapping CIs. The pooled effect estimate was close to zero, which suggested that the values of postoperative spherical equivalent were comparable between the toric and non-toric IOL groups.

### 3.8 Publication bias

**Figure 8:**
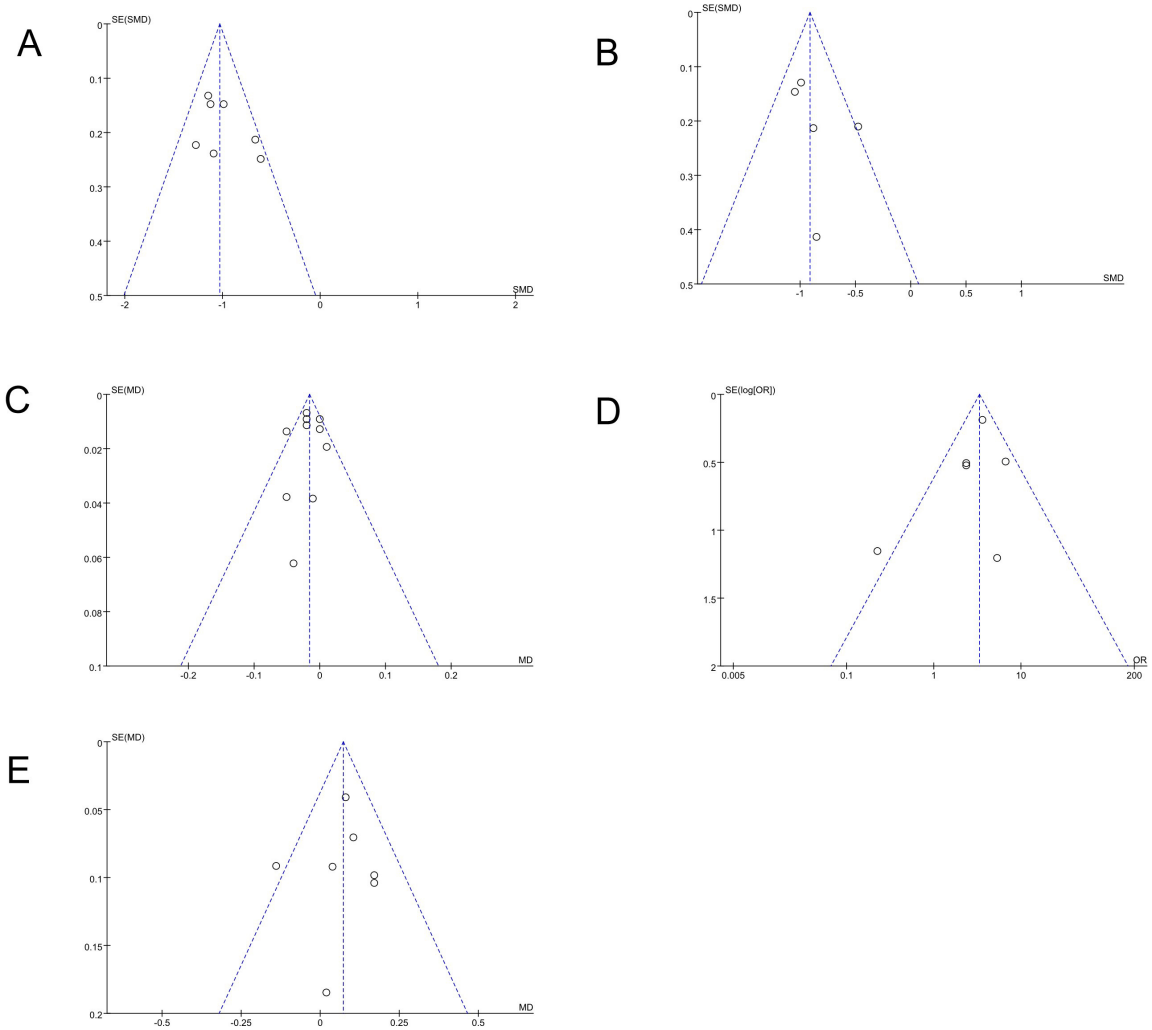
Funnel plots for assessed outcomes **(A)** Postoperative residual refractive astigmatism, **(B)** UDVA; **(C)** CDVA; **(D)** Proportion of eyes with residual refractive cylinder ≤ 0.5 D; **(E)** Postoperative spherical equivalent.

Publication bias was explored by visual inspection of funnel plots for the analyzed outcomes, including postoperative residual refractive astigmatism, uncorrected distance visual acuity, corrected distance visual acuity, the proportion of eyes with residual refractive cylinder of 0.50 D or less and postoperative spherical equivalent. These plots are shown in Figures 8A–E.

For each outcome, individual study estimates were scattered on both sides of the pooled effect, without a clear tendency for studies to cluster preferentially on one side of the funnel. No obvious asymmetry of the overall pattern of study distribution was observed.

The visual characteristics of the funnel plots were generally similar across outcomes, with studies distributed around the summary effect estimates rather than concentrated in a particular region. However, the limited number of studies available for some endpoints constrains the certainty of these qualitative assessments.

### 3.9 Sensitivity analysis

As an exploratory sensitivity analysis, the main meta-analyses were repeated after omitting each individual study in turn. These leave one out analyses did not materially change the direction or statistical significance of the pooled effects. This finding suggests that the overall results were not driven by any single study. Because only a limited number of studies contributed to each endpoint and most reports were judged to have low or moderate risk of bias, separate analyses restricted to studies at very low risk of bias were not performed. This is acknowledged as a limitation in the Discussion.

## 4 Discussion

Across the comparative clinical studies included in this meta-analysis, toric IOL implantation generally yielded lower postoperative residual refractive cylinder than non-toric IOL implantation in cataract patients with pre-existing corneal astigmatism. This pattern is in line with the refractive rationale that correcting corneal astigmatism at the IOL plane improves unaided distance visual acuity and reduces postoperative spectacle dependence. In this meta-analysis, these benefits were quantified, and the pooled estimates indicate a clear reduction in residual refractive astigmatism and a corresponding improvement in uncorrected distance visual acuity, while effects on corrected distance visual acuity and spherical equivalent were small or clinically modest (21).

The improvement in UDVA observed in our analysis is clinically intuitive, because UDVA is more sensitive than CDVA to residual refractive cylinder and small refractive errors. In practical terms, toric IOLs may allow more eyes to reach low levels of postoperative refractive cylinder and thereby achieve better unaided distance vision, without compromising corrected vision. Previous comparative cohorts and randomized trials have also shown that toric IOLs reduce postoperative refractive astigmatism and improve UDVA relative to non-toric monofocal lenses, especially when refractive accuracy and spectacle independence are key surgical objectives rather than best-corrected acuity alone (21, 25, 26). Our synthesis adds to this literature by providing updated, quantitative effect sizes for these outcomes based on contemporary studies. These observations align with the view of cataract surgery as a refractive procedure and with the increasing expectation of patients for good unaided distance vision after surgery.

In contrast, the observed difference in postoperative CDVA between toric and non-toric IOLs, although statistically significant, was very small in absolute terms and is likely to be of limited clinical relevance. CDVA is primarily constrained by macular status, optic quality, and neural visual potential; once refractive error is optimally corrected with spectacles, both toric and non-toric monofocal lenses would generally be expected to provide similar best-corrected acuity. This is consistent with clinical experience and with previous reports showing that toric IOLs primarily confer advantages in unaided performance and refractive precision rather than in raising the ceiling of corrected visual acuity (25). Clinically, this distinction is important for counseling: patients should understand that the benefit of toric IOLs lies in improved spectacle independence and refractive accuracy rather than in better “maximum” vision with correction.

The rate of eyes achieving a postoperative residual refractive cylinder ≤ 0.5 D is a valuable endpoint in refractive cataract surgery because it indicates the probability of achieving a clinically meaningful target of low astigmatism and reduced spectacle dependence. In our meta-analysis, toric IOL implantation was associated with a greater likelihood of achieving this refractive target compared with non-toric lenses. This finding is concordant with reports in special populations, such as older patients or those undergoing combined procedures, where toric IOLs have improved refractive outcomes and UDVA while maintaining low residual refractive cylinder (24, 26). In contemporary refractive cataract practice, such probability-based endpoints are often more intuitive for shared decision-making than mean differences alone, as they directly convey the proportion of patients expected to achieve a low-residual-astigmatism target after surgery.

Our results should also be interpreted in the context of alternative astigmatism-management approaches used in conjunction with non-toric IOLs, such as steep-axis clear corneal incisions or peripheral corneal relaxing incisions. Comparative work suggests that the incremental advantage of toric IOLs over these techniques tends to be more pronounced as baseline astigmatism increases, whereas differences can be smaller and more variable in lower-astigmatism subgroups (29). These findings support the view that toric lenses can provide relatively reliable and predictable astigmatic correction, while the size of the benefit relative to corneal procedures may be influenced by baseline cylinder, surgical technique, lens design and the duration of follow up. Especially in situations where the eyes have low preoperative astigmatism, the refractive gain obtained from toric IOLs may be limited, and patient preference and cost issues, as well as surgeon experience, should be factored in when choosing the best course of action.

Despite the merits brought by this meta-analysis, there are also several sources of heterogeneity that are relevant to clinical decision-making. Differences in baseline corneal astigmatism distributions, toric IOL models and haptic designs, alignment techniques, and follow-up intervals all have the potential to influence both the magnitude and the stability of toric IOL effects. Recent systematic reviews focusing on rotational stability have shown that lens model and design features can affect rotational behavior and, by extension, effective astigmatic correction (30). Moreover, evolving biometry technologies and toric calculators that incorporate posterior corneal astigmatism and effective lens position may further refine refractive predictability in ways that are not fully captured in older studies (16, 17, 31–33). These trends suggest that the benefits of toric IOLs may be even more consistent in current practice than in earlier eras, although longitudinal data remain needed.

Several limitations should be acknowledged. First, there was variation in baseline corneal astigmatism between studies, including differences in the lower and upper limits used for patient selection. Second, surgical techniques and perioperative strategies were not uniform, and aspects such as incision planning, the use of additional corneal relaxing incisions and the choice of lens model may have influenced both the magnitude and the stability of astigmatic correction. Third, biometric methods and intraocular lens power calculations were not fully standardized, particularly with respect to the treatment of posterior corneal astigmatism and estimates of effective lens position. Fourth, follow up duration varied, which may affect the assessment of rotational stability and long term refractive outcomes. Finally, patient reported outcomes and detailed measures of spectacle independence were not consistently reported and could not be synthesized, even though these aspects are important for refractive cataract surgery. In addition, the number of available studies for each endpoint did not permit separate analyses restricted to trials at very low risk of bias.

Despite these limitations, this meta-analysis enhances the current consensus regarding the indication of toric IOLs in eyes with pre-existing corneal astigmatism by pooling important refractive and visual outcomes from contemporary trials and cohorts. Instead of questioning clinical practice, the present results contribute to defining the extent of anticipated benefit and to identifying the clinical situations where the use of toric IOLs will most likely lead to significant enhancements in uncorrected visual function.

## 5 Conclusion

In eyes with cataract and pre-existing corneal astigmatism, toric intraocular lens implantation generally reduces residual postoperative refractive astigmatism and improves uncorrected distance visual acuity compared with non-toric lenses, although the effects on corrected distance visual acuity and spherical equivalent appear minimal. The higher proportion of eyes with residual refractive cylinder of 0.50 D or less further supports toric lenses as a useful refractive option when postoperative unaided distance vision is an important goal of surgery. By providing effect size estimates for key refractive and visual outcomes, this meta-analysis builds on current consensus and offers clinically useful evidence for patient selection and refractive planning, and underscores the need for further prospective investigations to define indication thresholds and long term rotational stability.

## Data Availability

The original contributions presented in the study are included in the article/supplementary material, further inquiries can be directed to the corresponding author.
